# Acute idiopathic gastric necrosis, perforation and shock

**DOI:** 10.4103/0974-2700.66564

**Published:** 2010

**Authors:** Nereo Vettoretto, Fabio Viotti, Lucio Taglietti, Maurizio Giovanetti

**Affiliations:** Department of General and Vascular Surgery, M. Mellini Hospital, Chiari (BS), Italy

Many causes have been described for acute gastric dilatation and necrosis (lifestyle habits, underlying morbidities, acute necrotizing inflammation, acute vascular insufficiency, post-operative complications). The onset of symptoms is generally catastrophic and surgical therapy must be emergent and aggressive because mortality is elevated for delayed diagnosis. We describe a case of sudden shock aroused after an orthopedic operation due to gastric ischemic rupture, apparently not ascribable to causes previously reported in the lay literature.

**Figure 1:**
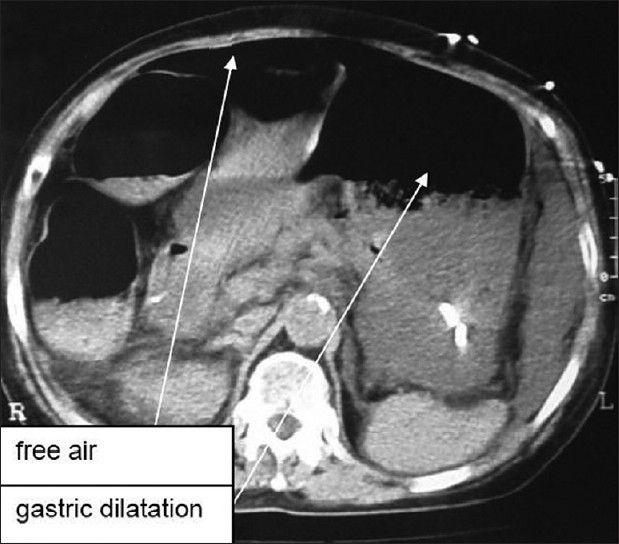
Computed tomography scan showing gastric dilatation and intraperitoneal free air

**Figure 2:**
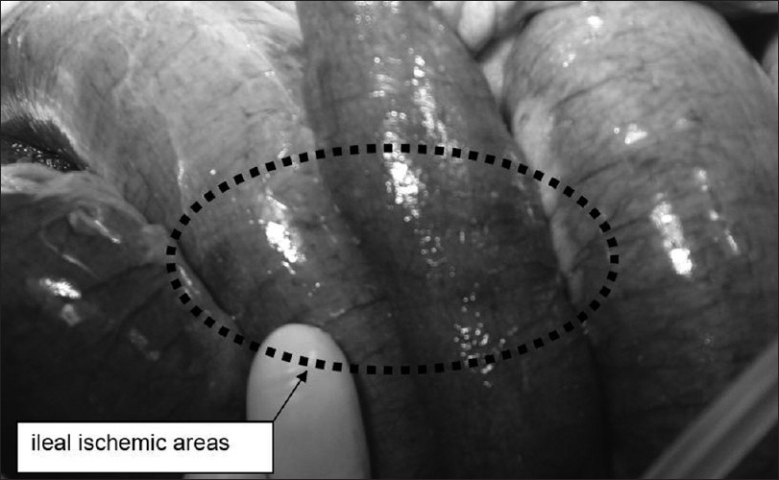
Intraoperative evidence of partial gastric necrosis and perforation

**Figure 3:**
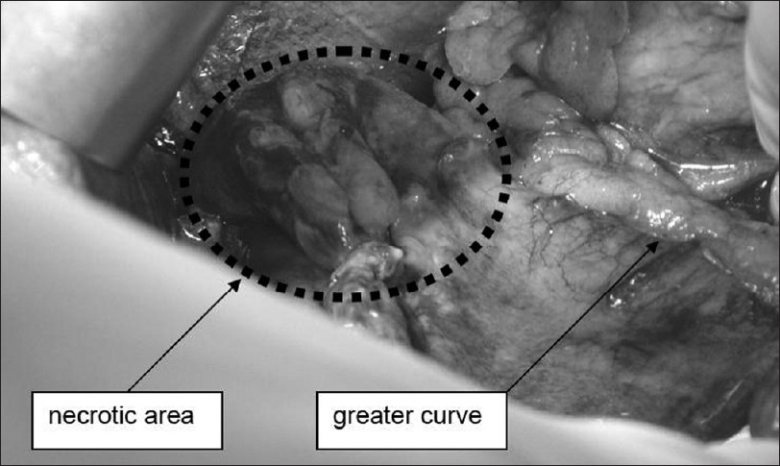
Intraoperative view of ileal ischemic spots

A 79-year-old woman underwent right hip replacement for femoral bone fracture. Past medical history was significant for hypertension, without any upper gastrointestinal pathology reported. In the first two post-operative (p.o.) days, there was no evidence of clinical or blood abnormalities apart from leukocytosis (white blood cell count, 17,350/mcl) and thus refeeding was started since the day after operation. Proton pump inhibitors were given in order to prevent stress ulcers or gastritis. On p.o. day 3, after a complaint of epigastric pain, a sudden hemodynamic shock manifested, with a blood pressure of 60/40, pallor, anuria, tachycardia and obnubilation, requiring fluid resuscitation and intubation for respiratory insufficiency. A nasogastric tube was positioned immediately after intubation. Physical examination evidenced hypertympanism with epigastric tenderness. No significant cardiac abnormalities were found on cardiogram and cardiac ultrasound (US); abdominal X-rays and US did not clarify the diagnosis while the computed tomography (CT) scan (performed after the fluid resuscitation and pressure values stabilized above 100 mmHg) evidenced a great gastric and intestinal dilatation, with diaphragm elevation and intraperitoneal free liquid and air [Figure 1]. Acute renal failure was noticed (creatinine 3.8 mg/dl). She underwent emergency laparotomy with a suspicion of mesenteric ischemia. Intraoperative findings were diffuse ischemic spots over most of the small bowel [Figure 2], together with a massive gastric dilatation complicated by focal full-thickness necrosis of the anterior wall [Figure 3]. No apparent pulse deficits were perceived in celiac trunk or superior mesenteric artery. The relatively small extension of gastric necrosis together with the fluid-resistant-shock were a contraindication to radical gastrectomy, and so we performed a “sleeve” gastrectomy to ablate the necrotic area of the anterior surface together with a mechanical emptying of the small bowel (with a double-lumen 18F Salem tube), a cecostomy and a feeding jejunostomy. Histological specimen revealed severe ulcerous-atrophic gastritis, with ischemic areas of necrosis. However, septic shock proved irreversible and the patient died on p.o. day 2, with the family declining autopsy.

Till date, more than 60 cases of gastric acute ischemia and perforation have been reported since its first description by Duplay in 1833, ascribing various causes, but this is the first report in the aftermath of orthopedic surgery. Gastric dilatation might be very important, so as to provoke abdominal compartment syndrome.[1] The stomach’s abundant vascular supply (right and left gastric, right and left gastroepiploic and short gastric vessels) renders acute ischemic necrosis a very rare disease. In fact, to be achieved in experimental animals, closure of the right and left gastric and gastroepiploic arteries together with at least 80% of the collaterals is required.[2] In the case described, a feeble pulse in the left gastric artery was perceivable, even in the hypotensive condition. When massive dilatation occurs, ischemia is presumably due to venous insufficiency: 14 mmHg of pressure is sufficient to impair venous outflow and intralumenal quantity of more than 3 L of fluid can distend the stomach to that pressure, although chronic distension of more than 15 L has been described in eating disorders (polyphagia, bulimia). Rupture can occur with intragastric pressures of more than 120 mmHg (corresponding to approximately 4 L of fluid), but pressure can reach these values even after cardiac compression during the course of resuscitation.[3] In our case, even if gastric insufflation can be hypothesized during emergency intubation maneuvers, no external cardiac massage has been performed, and shock and abdominal tension occurred previously. Distension and perforation can rapidly lead to neurogenic (vagal response) and subsequent septic shock. Atonicity of the stomach can also lead to massive distension when food intake is reprised, and this happens in case of anorexia nervosa or electrolyte imbalance. Mechanical factors can be implied in gastric dilatation, like bowel obstruction or pyloric stenosis,[4] and infectious causes (necrotizing gastritis) have been reported, generally involving immunocompromised patients (diabetes, AIDS, neoplasia).[5,6] Physiopathologic theories have been debated in the past: one advocated upper esophageal sphincter relaxation (due to debilitation or anesthesia) with consequent aerophagia and gastric dilatation as a cause. Atonic theory (muscular atrophy during prolonged starvation does not support rapid refeeding), superior mesenteric artery syndrome (vascular compression of the third duodenal portion) or functional diseases caused from regional alterations (in pancreatitis, ulcer and other abdominal inflammations) are other supposed etiologies. A consequence in events is postulated by Abdu: the first step should be mucosal necrosis, followed by full-thickness involvement of the gastric wall and perforation.[7] Clinically, emesis might be the initial symptom, but events can precipitate suddenly to shock, as reported in our case. Physical findings are abdominal distension and tympanism, with tenderness and peritonitis in case of perforation. Plain abdominal films and CT scan are useful in the diagnosis as they can demonstrate gastric distension and free air. Detension with nasogastric tube is mandatory as the first therapeutical act, followed by immediate surgery in case of perforation. Necrosis might be partial (mostly in the lesser curve due to vascular supply) or involving the full organ. Total gastrectomy is the procedure of choice, but it requires time and stable hemodynamic conditions. Mortality is very high, ranging from 50 to 80%.[8] Our patient was in advanced shock (bowel ischemic spots were probably due to prolonged hypotension), and could not stand total gastrectomy; thus, we performed the fastest possible operation. Partial resections have already been described in case of limited necrosis.[9] Small bowel emptying and cecostomy were performed in order to deflate the intestine. Feeding jejunostomy could be useful in case of post-operative fistulas. Unfortunately, irreversible septic shock provoked the death of the patient despite the prompt diagnosis and surgery. In this patient, shock established without any clinical symptoms or eating, infectious or cardiovascular disorders in past medical history and were probably due to multifactorial causes (previous short fasting, hypertensive vascular disease, intubation, recent surgery, possible undiagnosed thromboembolic accident), but none of them stands alone as an evident reason for the catastrophic event. As necropsy was not performed for parental denial, we can classify this as an idiopathic ischemic gastric infarction.[10] We can summarize the clinical steps that can be useful in case of p.o. shock in which we can suspect gastric ischemia and perforation [Table 1].

**Table 1:**
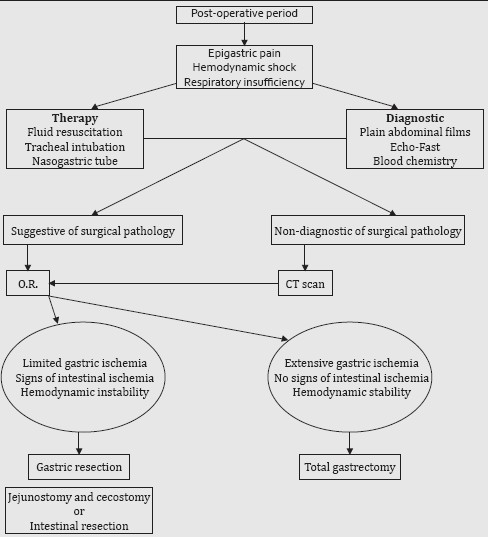
Flow chart in case of suspicion of gastric ischemia and perforation

We acknowledge that shock in the post-operative period might have many causes, one of which could be idiopathic gastric necrosis, especially in the older patient complaining of abdominal distension and pain, and prompt diagnosis and aggressive treatment are mandatory, but sometimes not sufficient to achieve success.
